# Low frequency of BRAF and KRAS mutations in Chinese patients with low-grade serous carcinoma of the ovary

**DOI:** 10.1186/s13000-017-0679-3

**Published:** 2017-12-22

**Authors:** Yan Xu, Rui Bi, Yaoxing Xiao, Xiaoyu Tu, Ming Li, Anqi Li, Ling Shan, Shuling Zhou, Wentao Yang

**Affiliations:** 1 Department of Pathology, Fudan University Shanghai Cancer Center, 270 Dongan Road, Xuhui District, Shanghai, 200032 China; 2 Department of Oncology, Shanghai Medical College, Fudan University, Shanghai, China; 3 Department of Pathology, Tongji University Shanghai East Hospital, 1800 Yuntai Road, Pudong New District, Shanghai, 200120 China

**Keywords:** Low-grade serous carcinoma (LGSC) of the ovary, KRAS mutation, BRAF mutation, Prognosis

## Abstract

**Background:**

Mounting evidence has shown that KRAS and BRAF are somatic mutations associated with low grade serous carcinoma (LGSC) of the ovary. However, the frequency of KRAS or BRAF mutation was variable in literatures, with a frequency of 16–54% for KRAS mutation and 2–33% for BRAF mutation. Meanwhile, the prognostic significance of KRAS or BRAF mutation remains controversial.

**Methods:**

Codons 12 and 13 of exon 2 of KRAS gene and exon 15 of BRAF gene were analyzed using direct Sanger sequencing in 32 cases of LGSC of the ovary. The associations between KRAS or BRAF mutation and clinicopathological characteristics, overall survival (OS) and disease-free survival (DFS) were statistically analyzed.

**Results:**

KRAS mutation was observed in nine cases (9/32, 28%) and BRAF mutation in two cases (2/32, 6%). KRAS and BRAF mutations were mutually exclusive. Neither KRAS nor BRAF mutation was statistically associated with OS or DFS in our cohort, although there was a favorable prognostic trend in patients with KRAS G12D mutation than those with KRAS G12 V mutation or wild-type KRAS for OS.

**Conclusions:**

The present study indicated a low frequency of BRAF or KRAS mutation in Chinese patients with LGSC of the ovary, and neither KRAS nor BRAF mutation is a prognostic factor.

## Background

Ovarian serous carcinoma is the most common ovarian malignancy. In 2004, Malpica et al. described a novel two-tier grading system to classify ovarian serous carcinoma as high-grade serous carcinoma (HGSC) or low-grade serous carcinoma (LGSC) [1]. In this system, a carcinoma is categorized-based primarily on the degree of nuclear atypia and secondarily on its mitotic rate. LGSC and HGSC are characterized by different clinicopathological and molecular features [1–5]. The two-tier grading system is now widely accepted and was adopted in the WHO classification system for female reproductive organ tumors (2014 version) [6]. Unlike HGSC, LGSC is more common in young patients and is associated with chemo-resistance and longer overall survival (OS). Typically, LGSC is also associated with KRAS and BRAF mutations that target specific cell signaling pathways [7–11].

Several reports have demonstrated that KRAS and BRAF mutations occur at a frequency of 16–54% and 2–33%, respectively, in ovarian LGSC [12–14]. The cohort in these studies included Caucasian and African individuals and a small cohort of individuals of other races. With the exception of a report by Cho that described 20 cases of Korean patients, few data are available regarding these mutations in Asian patients [15]. A previous study suggested that mutations in BRAF and KRAS contributed to the development of LGSC [16]. Patients with these mutations appear to have shorter survival [17]. Other reports have shown that BRAF or KRAS mutations in patients may be associated with an improved prognosis or favorable trends [12, 18, 19]. Therefore, the prognostic significance of KRAS and BRAF mutations remains controversial.

The purpose of this study was to evaluate the incidence of BRAF and KRAS mutations in patients with LGSC and to evaluate the prognostic significance of KRAS or BRAF mutations in LGSC in a Chinese population. In addition, we explore the association between KRAS and BRAF mutations and various clinical and pathological features.

## Methods

### Patients and samples

**Fig. 1:**
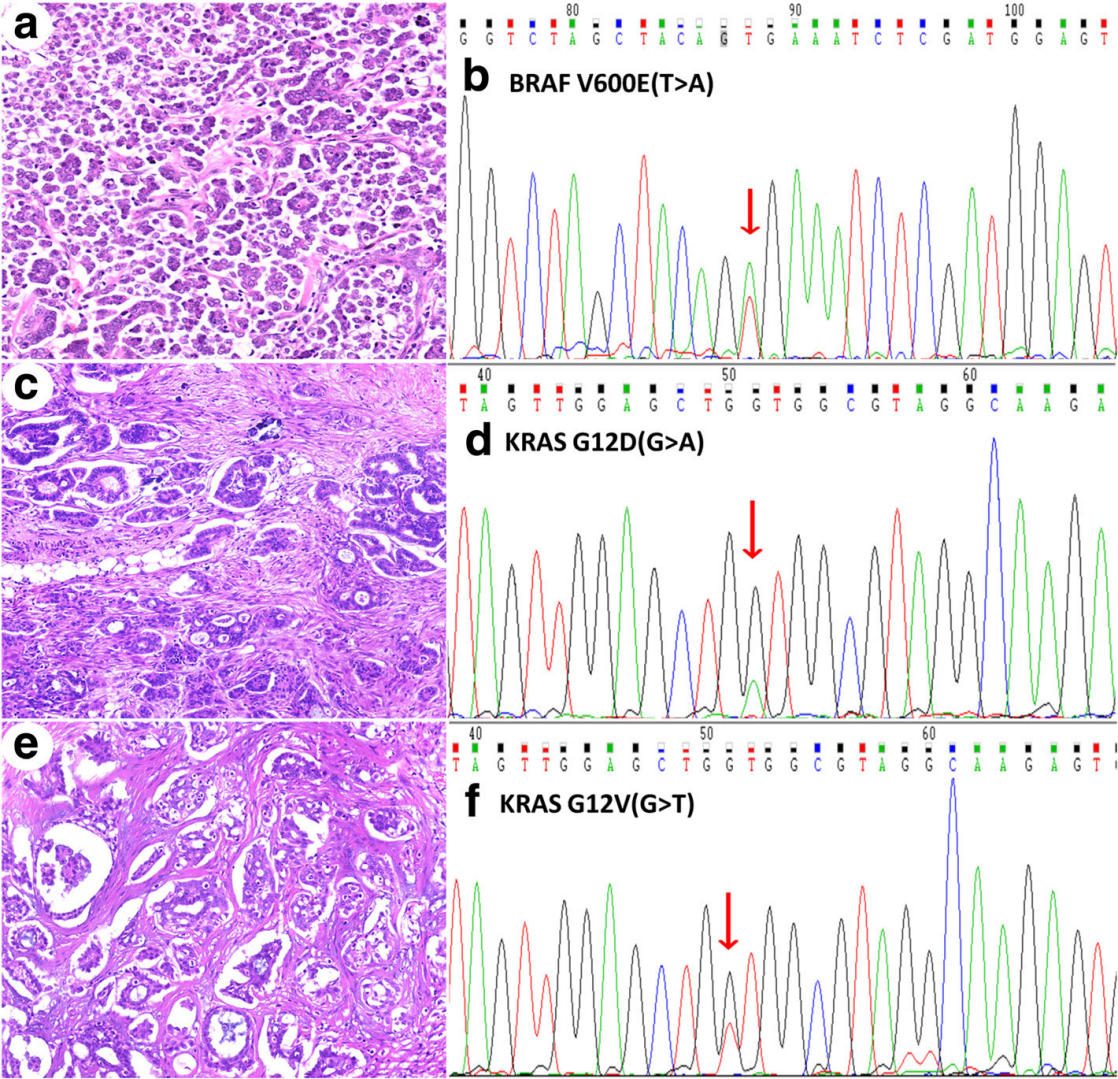
Histology and nucleotide sequences of KRAS or BRAF in 3 representative LGSC cases. Case 1: **a**, H&E staining of the tumor. **b**, Chromatogram of the nucleotide sequence shows a point BRAF V600E mutation (GTG to GAG) in LGSC. Case 2: **c**, H&E staining of the tumor. **d**, Chromatogram of the nucleotide sequence shows a point KRAS G12D mutation (GGT to GAT) in LGSC. Case 3: **e**, H&E staining of the tumor. **f**, Chromatogram of the nucleotide sequence shows a point KRAS G12 V mutation (GGT to GTT) in LGSC. H&E slides, magnification ×40

Thirty-two ovarian LGSC samples were collected from the Department of Pathology at Fudan University Cancer Hospital between February 2006 and September 2015. LGSCs were diagnosed according to the following criteria described by Malpica [1]: (1) low mitotic activity in <12/10 HPFs (high-power fields) and an absence of abnormal mitosis; (2) mild to moderate nuclear atypia with mostly uniform, round or oval nuclei and/or slightly irregular chromatin; (3) the presence or absence of a typical serous borderline tumor (SBT) component, and frequent psammoma bodies (Fig. 1). All cases were reviewed by two experienced gynecological pathologists (Yang WT and Bi R).

### DNA extraction and mutation analysis

We used polymerase chain reaction (PCR)-based direct gene sequencing to detect the KRAS (exon 2) and BRAF (exon 15) mutational status of each sample. Briefly, genomic DNA was extracted from FFPE tumor tissues using a Qiagen PCR purification kit (Qiagen, Valencia, CA, USA) according to the manufacturer’s instructions. Exon 2 of KRAS was amplified using PCR with the following specific primer pairs: 5’-TAAGGCCTGCTGAAAATGACTG-3′ (sense) and 5’-TGGTCCTGCACCAGTAATATG-3′ (anti-sense). The following specific primer pairs were used to amplify exon 15 of BRAF V600E: 5’-TCATAATGCTTGCTCTGATAGGA-3′ (sense) and 5’-GGCCAA AAATTTAATCAGTGGA-3′ (anti-sense). The following program was used to amplify both KRAS and BRAF: denaturation at 95 °C for 5 min followed by 40 cycles at 95 °C for 30 s, 54 °C for 30 s and 72 °C for 1 min. The PCR products were confirmed via agarose gel electrophoresis, purified using the DNA Clean/Extraction Kit (Gene Mark), and submitted to direct sequencing using the Big Dye Terminator Cycle Sequencing Kit (Applied Biosystems) according to the manufacturer’s instructions. The sequencing products were ethanol-precipitated before they were run on a 3700 genetic analyzer (Applied Biosystems), and the resulting sequence data were analyzed using Chromas software. Each mutation was verified in both the sense and anti-sense direction, and the results were independently evaluated by two investigators (Bi R and Xu Y).

### Statistical analysis

All patients were followed-up until Nov 30, 2015. OS was defined as the time from operation to either death or the last follow-up. Disease-free survival (DFS) was defined as the interval from the operation to disease recurrence or the last follow-up. DFS and OS were estimated using the Kaplan–Meier test for univariate analyses. A chi-square test was used to compare differences between groups. A *P*-value < 0.05 was considered to indicate statistical significance. Statistical analyses were performed using SPSS version 20.0 software (IBM, SPSS Statistics Armonk, NY, and USA).

## Results

### Clinical characteristics

The clinicopathological characteristics of the patients and the correlations with KRAS or BRAF mutation status are summarized in Table 1. The median age of the 32 patients was 55 years old (range, 21–77 years old). The median follow-up period was 26 months (range, 9–87 months). No statistically significant associations were found between KRAS or BRAF mutations and age, tumor size, laterality, cytology, FIGO stage, ascites, ovarian surface involvement, cancer antigen 125 concentration, metastases or lymph node involvement (all, *P* > 0.05) (Table 1).

**Table 1:** Clinicopathological characteristics and KRAS and BRAF mutations in LGSC

Clinicopathological characteristics	KRAS/BRAF gene		*P*-value
	Mutation (*n* = 11)	Wild-type (*n* = 21)	
Age (years)			0.106
< 45	1 (9.10%)	9 (42.86%)	
≥ 45	10 (90.90%)	12 (57.14%)	
FIGO stage			1.000
I/II	3 (27.27%)	7 (33.33%)	
III/IV	8 (72.73%)	14 (66.67%)	
CA125 (U/ml)			1.000
≤ 35 U/mL	0 (0.00%)	1 (4.76%)	
> 35 U/mL	11 (100.00%)	20 (95.24%)	
Tumor size(cm)			0.442
< 10	5 (45.45%)	6 (28.57%)	
≥ 10	6 (54.55%)	15 (71.43%)	
Laterality			0.053
Unilateral	7 (63.64%)	5 (23.81%)	
Bilateral	4 (36.36%)	16 (76.19%)	
Cytology			0.703
Negative	8 (72.73%)	13 (61.90%)	
Positive	3 (27.27%)	8 (38.10%)	
Ascites			0.441
Absent	2 (18.18%)	7 (33.33%)	
Present	9 (81.82%)	14 (66.67%)	
Ovarian surface involvement			0.815
No	3 (27.27%)	8 (38.10%)	
Yes	7 (63.64%)	11 (52.38%)	
Unknown	1 (9.10%)	2 (9.52%)	
Metastases			1.000
No	3 (27.27%)	7 (33.33%)	
Yes	8 (72.73%)	14 (66.67%)	
Lymph node involvement			0.392
No	1 (9.10%)	1 (4.76%)	
Yes	2 (18.18%)	1 (4.76%)	
Unknown	8 (72.73%)	19 (90.48%)	

### BRAF and KRAS mutations

Eleven of the samples in our cohort possessed mutations (two BRAF V600E, five KRAS G12D and four KRAS G12 V mutations). The remaining twenty-one samples contained no detectable mutations in these two genes. The mutation rates for KRAS and BRAF in the LGSC patients were 28.13% (9/32) and 6.25% (2/32), respectively. The KRAS mutations were located in codon 12 (100.00%, 9/9), and the mutation types were GGT > GAT (G12D) (55.56%, 5/9) and GGT > GTT (G12 V) (44.44%, 4/9) (Fig. 1). The BRAF mutations were located in codon 600 (100.00%, 2/2), and 2 cases of BRAF mutations were GTG > GAG (V600E) (Fig. 1). The two LGSC cases with BRAF mutations did not have KRAS mutations, indicating that these mutations are mutually exclusive.

### KRAS and BRAF mutations and patient survival

**Fig. 2:**
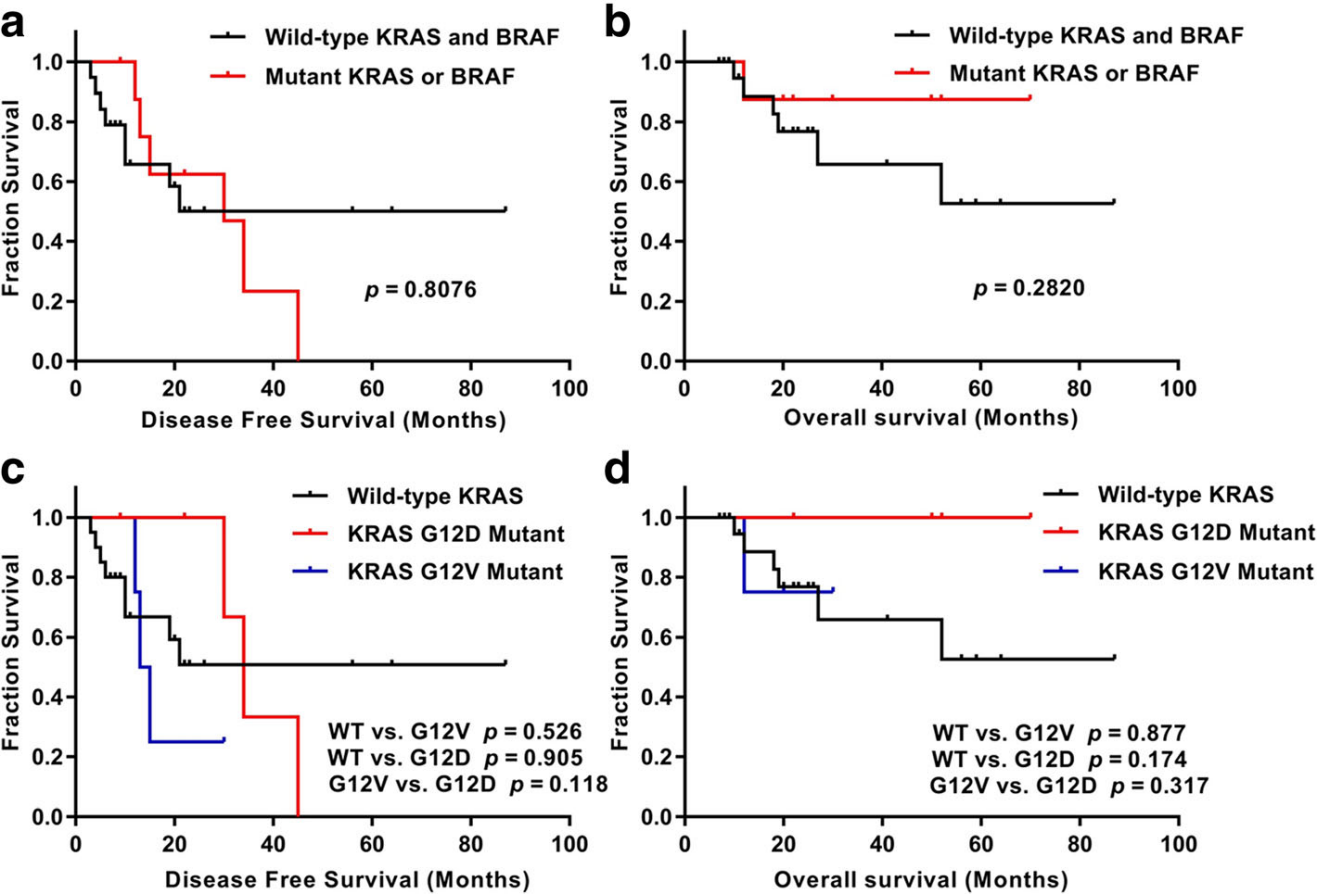
DFS and OS Kaplan–Meier analyses in ovarian LGSC. **a** and **b** No significant differences were found between patients with KRAS/BRAF mutations and those with wild-type KRAS/BRAF genes (*P* = 0.8076 for DFS, and P = 0.282 for OS). **c** and **d** Three KRAS mutation subtypes were not significantly correlated with DFS (**c**) or OS (**d**), but there was a favorable prognostic trend for KRAS G12D mutation compared with wild-type and G12 V mutation in OS (**d**)

There were no significant differences between wild-type and mutated KRAS/BRAF in DFS and OS (22 months vs. 30 months, *P* = 0.2820) (Fig. 2a and b). We also analyzed patient survival based on different KRAS mutation subtypes, including three groups: G12 V mutation, G12D mutation and wild type. No significant difference was observed in OS among patients with KRAS G12 V or G12D mutations and wild-type KRAS (*P* = 0.403). However, we observed that the median OS of patients with KRAS G12 V exhibited a decreasing trend compared with those with G12D (25 months vs. 50 months, *P* = 0.3173), and the same decreasing trend was found between the wild type and the G12D mutation (22 months vs. 50 months, *P* = 0.1742) (Fig. 2).

## Discussion

The frequencies of KRAS and BRAF mutations vary from 16 to 54% and 2–33%, respectively, in different reports in ovarian LGSC [12–14]. When whole-genome sequencing is applied, point mutations are much less common in LGSC of the ovary, and BRAF and KRAS are the most frequent mutations [13]. In our cohort, 28.13% (9/32) of the samples had KRAS mutations, while 6.25% (2/32) had BRAF mutations. This frequency was similar to that reported by Wong (19% KRAS and 2% BRAF) [12], Cho (30% KRAS and 10% BRAF) [15], Grisham (15.8% KRAS and 5.3% BRAF) [19], Farley (41% KRAS and 6% BRAF) [8] and Gershenson (22.8% KRAS and 3.8% BRAF) [18]. However, these results differed from the data published by Singer (26.7–54% for KRAS and 33% for BRAF) [16], which may be due to the small sample size in Singer’s study, which was only half the number of patients in our study. Nevertheless, BRAF mutation was a rare alteration in LGSC, and the frequency in most reports was lower than 10%.

In our cohort, we did not find a statistically significant difference in OS and DFS between patients harboring KRAS or BRAF mutations and patients with no KRAS or BRAF mutations. In addition, we combined cases of BRAF and KRAS mutations because there were only two cases with a BRAF mutation, and the outcome was similar to that of previous studies in that no statistically significant association was found between patients with tumors harboring KRAS or BRAF mutations and survival (Fig. 2a and b). Several reports have indicated that patients with KRAS or BRAF mutations may have a better prognosis than wild-type patients with ovarian LGSC [12, 18, 19]. Grisham et al. reported that the presence of a BRAF mutation in LGSC was associated with earlier stage disease and improved prognosis. Gershenson et al. reported that a KRAS or BRAF mutation may serve as a favorable prognostic factor and have a significant impact on outcomes in women with metastatic LGSC of the ovary or peritoneum. In our cohort, we also observed a favorable trend in prognosis with KRAS/BRAF mutation, even in the KRAS G12D mutation, but there was no significant difference. The possible explanations include selection bias due to limited sample sizes, geographic differences, ethnic heterogeneity, and short follow-up periods. Future studies with larger patient groups would provide more accurate information regarding these related links. Gershenson’s study included 79 LGSC cases diagnosed between 1975 and 2009 and had a long follow-up period. Similarly, there were 75 serous tumors (56 SBT and 19 LGSC) in Grisham’s study, with a median follow-up period of 35.9 months (0.8–129.3 months). However, the mean follow-up period was 30 months (9–87 months) in our cohort, which might not have been long enough to observe survival differences. Admittedly, this difference may be related to the smaller sample size in our study. In addition, typically, BRAF mutations have been more closely associated with SBT, whereas all the cases in our study were LGSC. Larger cohorts and longer follow-up periods will hopefully further clarify this issue. The results of targeting hotspot genes were limited compared with the results obtained by using a sequencing discovery phase for these two genes, indicating that it was highly likely that we missed additional case-specific mutations in our study population.

We observed that the prognostic significance of the KRAS mutation type was not significantly different, but there was a favorable trend toward better OS in patients with KRAS G12D mutation than in those with KRAS G12 V mutation or wild-type KRAS. This finding was similar to that of Tsang’s study [17], which reported that KRAS G12D mutation was associated with better OS in recurrent LGSC. We speculate that the sample size and shorter follow-up period is the bottleneck to obtaining significant differences. A similarly strong trend toward poor prognosis was also reported in lung adenocarcinoma with KRAS G12 V mutations [20].

KRAS and BRAF mutations are the most common mutations in LGSC, and both involve the mitogen-activated protein kinase (MAPK) pathway, as indicated by the effects of selective MEK inhibitors on recurrent or metastatic LGSC patients. However, no correlation was found between KRAS and BRAF mutations and therapeutic responses in the previously cited report [8]. With the development of next-generation sequencing technology, more abnormalities have been observed. These include NRAS mutations (26%), which occur at higher rates than either KRAS (21%) or BRAF (16%) mutations [21], and a 15-nucleotide deletion in the MAP2K1 gene, which encodes MEK1 [22, 23]. Hence, additional studies focused on mutations in LGSC should be performed.

## Conclusion

In summary, we have demonstrated a low frequency of KRAS or BRAF mutation in Chinese patients with LGSC of the ovary. Our data have indicated that BRAF mutation is a very rare event and KRAS mutation is more common than BRAF mutation in our cohort. Patients with KRAS G12D mutation may have a more favorable outcome trend than other patients, but no statistically significance identified. Further studies with large cohorts are necessary to determine the prognostic value of KRAS G12D mutation.

## Abbreviations

DFS: Disease-free survival
FFPE: Formalin-fixed, paraffin-embedded
FIGO: International Federation of Gynecology and Obstetrics
HGSC: High-grade serous carcinoma
LGCS: Low-grade serous carcinoma
OS: Overall survival
SBT: Serous borderline tumor
